# An evaluation of functional mental capacity in forensic mental health practice: the Dundrum capacity ladders validation study

**DOI:** 10.1186/s12888-018-1658-2

**Published:** 2018-03-27

**Authors:** Gearoid Moynihan, Ken O’Reilly, Jane O’Connor, Harry G. Kennedy

**Affiliations:** 1 National Forensic Mental Health Service, Central Mental Hospital, Dundrum, Dublin 14, Ireland; 2 Department of Psychiatry, Trinity College Dublin, Dublin 2, Ireland

**Keywords:** Schizophrenia, Mental illness, Functional capacity, Neurocognitive ability, Real world function

## Abstract

**Background:**

Because of the potential gravity of finding a person incompetent, assessment of mental capacity is challenging for clinicians. We aimed to test validity of a new structured professional judgement tool designed to assess functional mental capacity in three domains – finances, welfare and healthcare.

**Methods:**

Fifty-five male forensic psychiatric patients with Schizophrenia were interviewed using the Dundrum Capacity Ladders – a new semi-structured interview, and scores were assigned on a stratified scoring system, measuring ability to understand, reason, appreciate the personal importance of the decision at hand and communicate a decision. Data were also gathered pertaining to level of therapeutic security at the time of interview, diagnosis, neurocognitive function and a validated measure of real world function.

**Results:**

The results show that internal consistency and inter-rater reliability were high for all items. There were correlations between higher scores of functional mental capacity, neurocognitive function and measures of real world function in this population. Correlations were in the range 0.358 to 0.693, effect sizes that were moderate to high.

**Conclusions:**

The DUNDRUM Capacity Ladders appear to be a valid measure of functional mental capacity in this population. Further prospective studies of functional mental capacity as a measure of recovery are now required.

## Background

Functional Mental Capacity refers to neurocognitive and legal constructs concerning an individual’s ability to make and communicate decisions in an autonomous fashion. Its assessment has become increasingly important with the move away from paternalistic practises in healthcare provision towards an increased emphasis on an individual’s own treatment decisions (Schneider, 1998) [1]. It should also be considered as part of the assessment of functional outcomes in treating mental illnesses such as schizophrenia (Kahn & Keefe 2013) [2].

### The evolution of functional capacity

Functional Mental Capacity evolved first as a legal concept, arising from the interplay of expert evidence by clinicians and legal judgements and precedents, followed in some jurisdictions by legislation. The law identifies different competencies e.g. testamentary capacity, competency to marry, fitness to plead and stand trial. Decision-making capacity is but one element in the legal classification of competence. Decisional Capacity relates to a person’s ability to perceive, retain and understand information pertaining to the choices at hand, and to use this information to reason about and appreciate the consequences of the choice they may make. Capacity relates to a decision about a person’s capability to carry out a specific act or set of acts. Legal Competency relates to a decision made by a judge about whether a person, under the law, has or does not have the capability to carry out a specific act or set of acts. In this instance the clinician will provide the court with information summarising the person’s decision-making skills and offer an opinion on how these findings may affect the person’s abilities in a specific area. The judge then uses this information, together with legal factors to arrive at a finding of competence or incompetence.

A review of case law literature by Grisso and Appelbaum (1998) [3] resulted in the so-called ‘four abilities’ model in the assessment of functional mental capacity, namely; the ability to understand the information pertinent to the decision at hand, in keeping with the relevant facts of the decision; the ability to reason with this relevant information so as to engage in a logical weighing and comparison of the positive and negative consequences; the ability to appreciate the significance of the information for one’s own situation; and the ability to make and communicate a decision relating to the decision at hand.

Historically, legislative approaches to incapacitated persons were paternalistic. These approaches have been criticised as not being in keeping with the spirit of the Universal Declaration of Human Rights [4] and more explicitly in the United Nations’ Convention on the Rights of Persons with Disabilities, Article 3 (a) [5] which states: “*The principles of the present Convention shall be: Respect for inherent dignity, individual autonomy including the freedom to make one’s own choices, and independence of persons.”*

In most jurisdictions, modern legislation now includes specific statutory means of protecting the rights of people with mental disabilities, as seen in New Zealand, Ontario, Scotland and England and Wales. All assume presence of mental capacity unless established otherwise. However, all have slightly different definitions for the test of mental incapacity – some require the presence of mental disorder as a first step to define incapacity – as seen in Scottish law [6], and others view capacity under separable domains [7–9]. Newly published Irish legislation has also changed to a functional approach for capacity assessments [10]. In this article, we are primarily concerned with the clinical assessment of functional mental capacity. We regard this as a continuum from fully capable to seriously impaired, like any other neurocognitive mental ability or any physical ability. We are not primarily concerned with legal standards or thresholds for competence or incompetence.

### Difficulties in assessment of functional mental capacity

The assessment of functional mental capacity is one of the most ethically complex tasks which clinicians are called upon to perform on a routine basis. In every case a patient’s autonomy and right to self-determination must be weighed against a clinician’s judgment that as a consequence of illness, mental disorder or intellectual disability, they may not be capable of acting in their own best interest. Clinicians have an ethical and legal responsibility to demonstrate how an illness, mental disorder or intellectual disability may compromise an individual’s right to autonomy and self-determination when faced with a specific situation. However the reasons for doing this go beyond the assessment of legal competence. Clinicians must assess responses to treatment for many purposes. It is increasingly recognised that functional abilities are the most important outcomes of treatment for mental disorders such as schizophrenia [2], and functional outcomes are for practical purposes more important than symptomatic improvement. Neurocognitive abilities, social cognition and metacognition all play a part in influencing symptoms and functional outcomes (Fett et al. [11], Lam et al. [12], Gallagher & Varga [13], Lysaker et al. [14]). Indeed deficits in metacognition may be one of the distinguishing features between schizophrenia and other debilitating physical illnesses (Lysaker et al. [15]).

In practice the assessment of functional mental capacity can be complex and challenging. The complexity arises not only because of the balancing of responsibilities, but because functional mental capacity is inextricably linked with contextual and situational factors, and also because it is underpinned by a number of sequential cognitive process. In contrast to illnesses, mental disorders or intellectual disabilities, all of which may be static in nature, functional capacity is dynamic and refers to the extent to which patients can apply their abilities to a particular situation and context independently as well as when offered assistance. The cognitive processes which underpin functional capacity include comprehension, appreciation, reasoning, and judgment and difficulties may occur at any step of this process (Grisso and Appelbaum) [3].

The potential gravity of recommending that a court find a person incompetent – i.e. denying that person the right to an autonomous choice, means that assessments of functional mental capacity can be lengthy and onerous. In such an assessment, the clinician must take account of the patient’s mental illness (or otherwise), cognitive function and social attitudes, as well as collateral information from a reliable informant where possible. Furthermore, the information shared by the clinician during the assessment of functional capacity should be such that it is easily understood by the person whose mental capacity is being assessed. This holds true even when the assessment is not for a medico-legal purpose.

Although psychological science has produced objective measures of cognitive capabilities, which can be usefully applied to capacity evaluations, it would be wrong to determine capacity solely on the basis of cognitive testing. This is because cut-off scores for a particular individual faced with a particular competency question can never be determined in advance. Moreover, the results of cognitive assessments are proxies and thus one step removed from the ultimate issue of whether a patient can understand, reason, appreciate and make and communicate a decision based on the unique demands of a particular situation. All of these factors can be more holistically and directly assessed using clinical interviews with the patient and informants. However, in contrast to cognitive assessments which have established psychometric properties, the reliability and validity of unstructured clinical interviews is unknown and unmeasurable.

Research by Volicer and Ganzini [16] showed that psychiatrists, psychologists and geriatricians do not adhere to a uniform approach for the purpose of assessments of functional capacity. Their work supports the idea that physicians’ competence assessments are often subjective and inconsistent (Marson, McInturff et al.) [17].

Case law [18, 19] internationally has increasingly placed emphasis on the patient’s right to all material information pertaining to a particular proposed treatment, in order to make an informed decision. However, if a person is suffering from a psychotic illness, it is foreseeable that sharing too much information about treatment options may overburden the person’s capacities to make an informed treatment decision, as demonstrated by Kennedy, Dornan et al. [20]. It follows that decision-making in other domains would be similarly affected. Furthermore, other illnesses such as dementia would have similar deleterious effects on decision-making ability.

There are a number of structured professional judgement tools which have been developed for assessment of functional mental capacity, the majority of which are based in Decision Theory [21]; that is identifying the values, uncertainties and other issues relevant in a given decision, its rationality, and the resultant optimal decision. Perhaps the best-known of these in a forensic arena are the MacArthur Competence Assessment Tools for Consent to Treatment [22] and Fitness to Plead [23]. In a psycho-geriatric setting, other tools have also been devised, e.g. The Hopkins Competence Assessment Tool [24]. Fazel, Hope and Jacoby [25] have also published a structured approach to the assessment of mental capacity to complete advance directives, emphasising a patient centred approach. A key feature of these assessment tools is that they provide clinicians with a structure for carrying out their own interviews thus improving transparency, reliability and objectivity. For example, in addition to using a validated functional capacity interview in a complementary and concurrent fashion, clinicians can use capacity instruments as a template to structure their own judgment by substituting the specific idiosyncratic question facing their patient into the structure of the instrument. Used in this manner a structured professional judgement approach to assessing functional mental capacity capacities could facilitate clinician’s ability to communicate their opinions in legal settings and to reduce the potential for idiosyncratic differences between experts. More importantly, the assessment of functional mental capacity as a treatment outcome can become a reliable and valid measure. One recent study employed the MacArthur instruments as outcome measures in a controlled trial of cognitive remediation therapy for psychosis (Naughton et al. 2012) [26]. In addition to improved transparency of clinical decision-making, functional capacity instruments are useful to identify targets for treatment and to measure change. The extent to which functional mental capacities change over time is of great importance when considering the legal and human rights protections necessary for mentally incapacitated patients, including those detained and treated under mental health legislation.

Although functional capacity assessments are often specific to the decision in hand, in practice clinicians are most often required to make decisions regarding a patient’s capacity to manage their finances, to consent to medical treatment and to make welfare decisions concerning living independently. Currently there is no single instrument to assist clinicians when assessing functional capacity in these three domains. No single instrument has a framework that is readily transferable to idiosyncratic situations. Because of the routine nature of these tasks a valid and reliable functional capacity assessment in these domains would be particularly useful to help clinicians structure their judgments.

In examining these tools, we noted the need for a different form of rating of functional mental capacity, in order to take account of its continuous rather than binary form.

## Methods

### Objectives

We aimed to establish validity for a structured professional judgement tool to assess functional mental capacity in three domains – finances, welfare and healthcare. In this paper we present results of inter-rater reliability, construct validity (in the form of internal consistency) and criterion validity as compared to neurocognitive function. We wanted to rate each of these using a common decision-making structure for understanding, reasoning, appreciation and decision-making. We wanted these to be structured in such a way that even the most severely ill could be rated and that ratings were on a continuous scale rather than a dichotomous one. We have also examined the relationships between this measure of functional mental capacity, real world function and neurocognitive function.

We hypothesised that patients currently cared for in more highly structured and supportive hospital environments of higher therapeutic security would score lower in terms of functional mental capacity than those who had progressed further along the therapeutic recovery pathway within the hospital. We also hypothesised that scores of functional mental capacity would correlate with scores of occupational, social and symptomatic function; and neurocognitive function.

### Setting

The Central Mental Hospital provides high, medium and low therapeutic security and community follow-up mental health services for a population of 4.6 million [27]. At the time of the study there were eighty-two male in-patient beds at varying levels of therapeutic security, and sixteen patients under community supervision. Patients can be admitted to the hospital from prisons under the Criminal Law (Insanity) Act 2006 [28], if medically certified. They can also be admitted from the courts if found unfit to stand trial or not guilty by reason of insanity. Additionally, the service provides a tertiary referral and assessment service for local community mental health teams and admits patients under the civil Mental Health Act 2001 [29] on transfer from local psychiatric hospitals.

Male patients in the Central Mental Hospital are admitted to a high secure admission ward. From there they can move to a series of medium secure units and finally to low secure and pre-discharge units (Pillay et al. 2008) [30]. This is a patient-centred approach, as each service-user is placed at an appropriate level of therapeutic security according to their individual need. These placements correspond to levels of risk, symptom severity and the patient’s overall level of functioning. Location at the time of assessment was ranked according to the level of therapeutic security – high, medium or low. This is an ordinal ranking according to the staff-to-patient ratio, and the ranking system matches the position of each unit along the therapeutic pathway as service-users progress from admission to discharge. The units are arranged in clusters by level of therapeutic security – acute cluster (high secure), medium cluster (medium secure) and rehabilitation and recovery cluster (low secure) [30].

### Study design

Three vignettes were drafted in simple language to assess functional mental capacity in three domains – finances, welfare and healthcare. The vignettes were designed to assess patients’ abilities to: understand and retain the information relevant to the dilemma in each vignette, reason through the dilemma, appreciate the need for a decision and make and communicate a decision in each vignette.

A stratified scoring system from zero to one hundred was designed for each of the four domains understanding, reasoning, appreciation and ability to make and communicate a decision with definitions tethering the ratings at every ten points so that scores could be assigned for each of the three vignettes.

The vignettes were then reviewed by three focus groups, including senior nursing staff, senior social work staff, occupational therapy, consultant forensic psychiatrists, clinical psychologists and patient advocates, and suggested alterations were made accordingly.

Ethical approval for the study was sought from and granted by the Research Ethics, Audit and Effectiveness Committee of the Central Mental Hospital.

Patients were given written information about the study, and all participants who were assessed by their treating psychiatrists as capable of giving consent gave written informed consent to participate.

### Inclusion & Exclusion Criteria

All male patients with a diagnosis of schizophrenia or schizoaffective disorder using Structured Clinical Interview for Schizophrenia (Structured Clinical Interview for DSM-IV (SCID; First et al. 2002 [31]). resident in the Central Mental Hospital or under community supervision by the forensic recovery and rehabilitation service during the study period (January 2013 – January 2015) were considered eligible to participate. Female patients were excluded as this group makes up a very small proportion of forensic mental health service-users in this jurisdiction. Male patients who did not have English as a first language were excluded, as were those who were deemed too behaviourally disturbed to participate or unable to give consent as assessed by their treating consultant psychiatrists.

### Data collection

In order to rate the Dundrum Capacity Ladders (DCL), GM interviewed all patients who met inclusion criteria and consented to participate. Their responses were recorded in writing, and assigned a score on the ladder scoring system. For measures of inter-rater reliability, GM and JOC took a random sample of completed interviews and scored them using the DCL system. Data regarding contemporaneous level of therapeutic security, diagnosis as confirmed by treating consultant psychiatrist, and a validated measure of function, scored by the patients’ treating consultant psychiatrist, the MIRECC-GAF [32] was also recorded by the primary researcher. GM and JOC were blind to the ratings of the MIRECC-GAF and MCCB.

As the MIRECC-GAF and Dundrum Capacity Ladders have different maximum scores (300 and 1200 respectively), the total mean scores of each were corrected to a common total, 100.

### Cognitive assessment

Cognitive functioning was assed using the MATRICS Consensus Cognitive Battery [33] (MCCB) by Masters level assistant psychologists trained in its use. The MATRICS battery covers seven cognitive domains: processing speed; attention/ vigilance; working memory; verbal learning; visual learning; reasoning and problem solving and social cognition (assessed using social reasoning tasks for understanding and managing emotions taken from the ‘managing emotions’ subtest of the Mayer-Salovey-Caruso Emotional Intelligence Test (MSCEIT) which is a social reasoning test). The test comprises of vignettes of various situations, and options for coping with the emotions depicted in these vignettes. Participants are required to indicate the effectiveness of each solution ranging from one (very ineffective) to five (very effective). Overall performance on the MCCB is measured using a composite score which is the preferred outcome for treatment studies. Because there is growing awareness that non-social and social cognition are separable dimensions, the MCCB scoring system now provides an option for a neurocognitive composite that does not include the social cognition sub-scale. In validation studies, and in antipsychotic trials of stable patients, the MCCB demonstrated excellent reliability, minimal practice effects and significant correlations with measures of functional capacity. Those rating the MCCB were blind to the ratings of the Dundrum Capacity Ladders and also blind to the ratings of the MIRECC-GAF.

### Statistics

All data was entered using SPSS-22. Internal Consistency was assessed using Cronbach’s Alpha. Inter-rater Reliability was assessed using Spearman’s Rho and the Intra-class Correlation Coefficient. The relationships between Capacity Scores, function (MIRECC-GAF), Level of Therapeutic Security, age, length of stay and neurocognitive ability were assessed using Spearman’s Rho for non-parametric correlations. We took r values of 0.1 to 0.3 as small effect sizes, 0.3 to 0.5 as moderate effect sizes and r values greater than 0.5 as large effect sizes [34]. The relationship between Dundrum Capacity Ladder scores, MCCB scores and GAF scores was examined using regression analysis.

## Results

Fifty-five patients gave written consent to participate and completed the interviews with the primary researcher**.** The mean age of participants was 40.5 years (range 22–73 years). The mean length of stay from admission to the hospital to date of assessment was 2029 days (range 2–14,638 days). Using SCID-I, Forty-eight patients met DSM-IV-TR criteria for Schizophrenia, and seven for Schizoaffective Disorder. The corrected mean MIRECC-GAF was 51.43.

### Validity measures

#### Internal consistency

Cronbach’s alpha measures the internal consistency of a scale, the extent to which each item of a scale correlates with the overall score. Cronbach’s alpha was high for each of the three domains: Finances 0.960, Welfare 0.973 and Healthcare 0.973. It was similarly high for each of the four abilities measured: Understanding 0.964, Reasoning 0.979, Appreciation 0.968 and Decision 0.961.

Table 1 shows that when omitted, only the Appreciation items on each of the vignettes lead to a small increase in the Cronbach’s alpha score. No item, if omitted, lead to a significant increase in the alpha statistic. When comparing abilities across the vignettes, no item if omitted lead to a significant increase in alpha score.

**Table 1:** Internal Consistency for the subscales of each of the three domains, and for the abilities across the Dundrum Capacity Ladders, *N* = 55

Across Domains	Subscale total alpha	Corrected item – Total Correlation	Cronbach’s Alpha if item deleted	Across Abilities	Subscale Alpha	Corrected item – Total Correlation	Cronbach’s Alpha if item deleted
Finances	0.960	–	–	Understanding	0.964	–	–
FU	–	0.948	0.933	FU	–	0.892	0.970
FR	–	0.926	0.942	WU	–	0.951	0.928
FA	–	0.812	0.974	HU	–	0.931	0.943
FD	–	0.941	0.935	Reasoning	0.979	–	–
Welfare	0.973	–	–	FR	–	0.934	0.983
WU	–	0.950	0.959	WR	–	0.967	0.960
WR	–	0.941	0.962	HR	–	0.962	0.960
WA	–	0.892	0.976	Appreciation	0.968	–	–
WD	–	0.953	0.958	FA	–	0.893	0.981
Healthcare	0.973	–	–	WA	–	0.959	0.933
HU	–	0.949	0.960	HA	–	0.947	0.942
HR	–	0.937	0.963	Decision	0.961	–	–
HA	–	0.900	0.975	FD	–	0.883	0.967
HD	–	0.956	0.957	WD	–	0.922	0.938
–	–	–	–	HD	–	0.946	0.920

#### Inter-rater reliability

Thirteen interview transcripts were chosen at random and scored independently by the primary and secondary researchers on the Dundrum Capacity Ladders. Inter-rater reliability was assessed using Spearman’s rho and the Intra-class Correlation Coefficient. These results are shown in Table 2.

**Table 2:** Inter-rater reliability: Spearman’s rho and Intra-class Correlation Coefficients for each subscale. *N* = 13 patients, two raters

Domain	Spearman’s Rho	Intra-Class Correlation Coefficient (95% CI)
Finances		
FU	0.930	0.975 (0.921–0.992)
FR	0.969	0.998 (0.992–0.999)
FA	0.957	0.977 (0.925–0.993)
FD	0.928	0.922 (0.765–0.975)
FT	0.979	0.989 (0.966–0.997)
Welfare		
WU	0.967	0.991 (0.972–0.997)
WR	0.963	0.995 (0.985–0.999)
WA	0.991	0.991 (0.971–0.997)
WD	0.938	0.983 (0.946–0.995)
WT	0.974	0.998 (0.992–0.999)
Healthcare		
HU	0.917	0.988 (0.960–0.996)
HR	0.950	0.985 (0.952–0.995)
HA	0.973	0.972 (0.910–0.991)
HD	0.938	0.983 (0.945–0.995)
HT	0.914	0.992 (0.973–0.997)
DCL Total	0.964	0.995 (0.982–0.998)

All correlations significant at the 0.01 level (2-tailed)

### Relationship between measured capacity scores and neurocognitive ability

Forty-one patients’ neurocognitive and social cognitive abilities were assessed using the MATRICS Consensus Cognitive Battery. Spearman’s rho was assessed for non-parametric correlation between subscales of the neurocognitive battery, length of stay, age, MIRECC-GAF, and the three subscales of the DUNDRUM Capacity Ladders in a post-hoc analysis. Length of stay did not correlate with cognitive function. Age was found to correlate inversely with processing speed and reasoning. The GAF and all its sub-scales correlated with each of the domains of neurocognitive function. This relationship is shown in Table 3.

**Table 3:** Relationship between MATRICS Sub-scales, Length of stay, age and GAF MIRECC, independently rated. Spearman’s rho. *N* = 41

	Length of Stay	Age	GAF OCC	GAF Soc	GAF Sym	GAF Tot
PS	−0.018	−0.375 *	0.494 **	0.317 *	0.326 *	0.429 **
An	− 0.206	−0.274	0.539 **	0.416 **	0.442 **	0.508 **
WM	− 0.020	−0.137	0.640 **	0.471 **	0.453 **	0.575 **
VeL	0.018	−0.244	0.626 **	0.462 **	0.394 *	0.546 **
ViL	0.142	−0.185	0.529 **	0.399 **	0.320 *	0.469 **
Rg	− 0.024	−0.401 **	0.506 **	0.318 *	0.332 *	0.442 **
SC	− 0.269	−0.107	0.558 **	0.508 **	0.599 **	0.619 **
MC	− 0.056	−0.274	0.673 **	0.515 **	0.503 **	0.626 **
NCC	− 0.037	−0.251	0.631 **	0.472 **	0.430 **	0..567 **

**Correlation is significant at the 0.01 level (2-tailed)

*Correlation is significant at the 0.05 level (2-tailed)

There were significant correlations between the Dundrum Capacity Ladders sub-scales and those in the MATRICS battery. This finding was replicated when examining the relationship between the MIRECC-GAF, its sub-scales and the Dundrum Capacity Ladders, as shown in Table 4. All correlations significant at the 0.01 level were in the range *r* = 0.358 to 0.693, effect sizes that were moderate to large.

**Table 4:** Relationship between MATRICS sub-scales, MIRECC-GAF, and sub-scales of Dundrum Capacity Ladders, independently rated. Spearman’s rho

Domain	PS	An	WM	VeL	ViL	Rg	SC	MC	NCC	GAF Occ	GAF Soc	GAF Sym	GAF Tot
N	41	41	41	41	41	41	41	41	41	55	55	55	55
Finances													
FU	0.473 **	0.505 **	0.509 **	0.545 **	0.564 **	0.516 **	0.381 *	0.610 **	0.600 **	0.594 **	0.540 **	0.463 **	0.589 **
FR	0.439 **	0.474 **	0.518 **	0.538 **	0.578 **	0.460 **	0.418 **	0.603 **	0.587 **	0.641 **	0.586 **	0.500 **	0.630 **
FA	0.210	0.333 *	0.338 *	0.373 *	0.322 *	0.357 *	0.145	0.363 *	0.374 *	0.541 **	0.491 **	0.358 **	0.498 **
FD	0.418 **	0.491 **	0.525 **	0.507 **	0.507 **	0.450 **	0.339 *	0.574 **	0.579 **	0.616 **	0.521 **	0.418 **	0.560 **
FT	0.444 **	0.518 **	0.540 **	0.550 **	0.560 **	0.486 **	0.368 *	0.610 **	0.606 **	0.618 **	0.569 **	0.418 **	0.611 **
Welfare													
WU	0.505 **	0.510 **	0.532 **	0.549 **	0.569 **	0.603 **	0.458 **	0.644 **	0.632 **	0.626 **	0.528 **	0.460 **	0.612 **
WR	0.494 **	0.512 **	0.558 **	0.522 **	0.514 **	0.548 **	0.482 **	0.631 **	0.615 **	0.670 **	0.625 **	0.526 **	0.663 **
WA	0.285	0.300	0.336 *	0.349 *	0.404 **	0.386 *	0.240	0.405 **	0.410 **	0.567 **	0.529 **	0.371 **	0.530 **
WD	0.483 **	0.512 **	0.561 **	0.534 **	0.463 **	0.578 **	0.433 **	0.615 **	0.604 **	0.687 **	0.577 **	0.468 **	0.631 **
WT	0.503 **	0.503 **	0.547 **	0.548 **	0.529 **	0.586 **	0.449 **	0.634 **	0.625 **	0.664 **	0.620 **	0.492 **	0.648 **
Healthcare													
HU	0.498 **	0.514 **	0.550 **	0.561 **	0.599 **	0.570 **	0.489 **	0.656 **	0.638 **	0.661 **	0.581 **	0.480 **	0.635 **
HR	0.485 **	0.509 **	0.529 **	0.535 **	0.549 **	0.534 **	0.506 **	0.637 **	0.620 **	0.663 **	0.601 **	0.496 **	0.642 **
HA	0.294	0.426 **	0.503 **	0.476 **	0.501 **	0.361 *	0.329 *	0.505 **	0.500 **	0.667 **	0.575 **	0.448 **	0.613 **
HD	0.442 **	0.500 **	0.521 **	0.533 **	0.527 **	0.481 **	0.450 **	0.598 **	0.589 **	0.637 **	0.573 **	0.492 **	0.623 **
HT	0.501 **	0.533 **	0.570 **	0.572 **	0.558 **	0.562 **	0.504 **	0.659 **	0.642 **	0.693 **	0.610 **	0.506 **	0.660 **

** Correlation is significant at the 0.01 level (2-tailed)

*Correlation is significant at the 0.05 level (2-tailed)

### Relationship between measured capacity scores, level of therapeutic security and scores of function

The relationship between current place on the recovery pathway from acute / high security to medium security and minimum security was tested using ANOVA (Table 5). Post-hoc Bonferroni corrections showed that the mean GAF for the minimum security rehabilitation and recovery cluster patients was significantly better than the acute cluster patients, while for the DCL, each cluster differed significantly from each of the other clusters. Age did not differ significantly, but length of stay did differ as expected. The mean total GAF score and mean total DCL scores differed significantly, with mean DCL scores differing significantly at each step. It is notable that in the acute cluster, patients were severely impaired (GAF mean = 47.3 S.D. =16.2, DCL mean = 18.6 S.D. =26.1) while those in the minimum security / rehabilitation and recovery stage functioned significantly better (GAF mean = 72.7 S.D. =14.2, DCL mean = 78.8 S.D. =21.1).

**Table 5:** Distribution of patient characteristics, GAF total score and DUNDRUM Capacity Ladder total score across the recovery pathway

Variable	Acute high secure		Medium secure		Minimum secure R&R		ANOVA	
n	17		27		11			
	mean	SD	mean	SD	mean	SD	F	p
Age (years)	38.6	10.3	40.6	10.7	43.1	14.2	0.5	0.6
Length of stay (days)	590.7	1686.9	2201.9	2715.6	3990.0	3767.9	5.4	0.008
GAF total (0–100)	47.3	16.2	46.5	11.1	72.7	14.2	16.4	0.001
DCL total (0–100)	18.6	26.1	50.9	20.3	78.8	21.1	25.3	0.001

## Discussion

The findings of this study demonstrate the reliability and validity of a new measure of functional capacity, the Dundrum Capacity Ladders, in a national cohort of male forensic mental health patients with schizophrenia or schizoaffective disorder. Within this sample consisting of patients at different levels of therapeutic security as well as differing stages of treatment, the Dundrum Capacity Ladders showed excellent internal consistency and interrater reliability. Specifically, Cronbach’s alpha for the total score on all three domains was greater than 0.9, and the Inter-class correlation coefficient for the total scores was also greater than 0.9. Moreover, Dundrum Capacity Ladders demonstrated excellent convergent validity with the MATRICS Consensus Cognitive Battery (MCCB) for cognitive deficits in schizophrenia. The correlations between the composite score of the MCCB and the total scores for finance, welfare and health domain where are all significant and large. Significant and large correlations were also observed between Dundrum Capacity Ladders and the Global Assessment of Functioning (GAF) in relation to its occupational, social and symptomatic domains. In other words, amongst forensic patients with schizophrenia and schizoaffective disorder, those who had higher levels of functional capacity, performed better on a separate assessment of occupational and social functioning and were judged to be less symptomatic. Finally, and as would be expected, patient performance on the Dundrum Capacity Ladders was significantly different across three levels of therapeutic security, with those patients in lower levels of therapeutic security possessing greater levels of functional capacity.

The size of the correlations between the Dundrum Capacity Ladders and the MCCB highlights the fundamental relationship between cognitive ability and functional capacity in the areas of understanding, appreciation, reasoning and judgment. In the cases of the financial, welfare, and health domain the MCCB composite score could account for approximately 40% of the variance. This finding suggests that functional mental capacity which is typically considered to be a dynamic construct may not be as separable from cognitive functioning, typically considered a static construct, as previously believed. The magnitude of these correlations are considerably greater than what has previously been observed (Moser et al.) [35]. But consistent with previous research psychiatric symptoms as measured by the GAF were also strongly correlated with functional capacity (Dornan et al.) [36]. However, whereas psychiatric symptoms can be treated effectively with appropriate medication and a therapeutic environment, the cognitive deficits experienced by patients with schizophrenia are less easily ameliorated (Keefe et al.) [37]. Although some improvement may be possible using psychological approaches like cognitive remediation therapy, and by pharmacological approached such as minimising anticholinergic burden (O’Reilly et al. [38]; O’Reilly et al. [39]).

The Dundrum Capacity Ladders are likely to have a number of practical applications. To begin with, the ladders provide clinicians with a useful platform to structure their judgments when determining functional mental capacity to manage finances, to consent to treatment, and to choose to live independently. Clinicians can choose to administer the vignettes or also to use the ladders to structure their own professional judgment regarding patient capacity. The Dundrum Capacity Ladders can also be used as routine outcome measure for psychiatric and psychological interventions, which is in keeping with the current emphasis on functional outcomes with schizophrenia research and treatment [40, 41]. As a measure of recovery the Dundrum Capacity Ladders may also be useful for guiding psychological interventions. A focus on measuring an improving patient capacity is particularly important within contexts where patients are involuntary detained and whether their human rights are by necessity compromised.

The study has a number of strengths as well as limitations. To begin with the study consisted of a national forensic mental health cohort with schizophrenia and schizoaffective disorder. In addition, convergent validity was also established with a consensus measure of cognitive ability the MCCB whose psychometric properties are established. The participants in this study were at different stages of treatment and under different levels of therapeutic security, demonstrating that the Dundrum Capacity Ladders are applicable to a range of patients. Weaknesses of the study include that the sample consisted entirely of male patients, hence generalisability to female patients may be questioned. Additionally, no attempt was made to determine concurrent validity using established measures of functional capacity such as the MacArthur scales, or criterion validity such as predicting the outcome of legal processes. Also it remains unclear the extent to which the Dundrum Capacity Ladders are sensitive to change. Future research should concentrate on addressing these limitations.

## Conclusions

We have concluded that the Dundrum Capacity Ladders are a valid tool for measuring functional mental capacities in a severely ill population with schizophrenia. Furthermore, we believe that our results highlight the inter-related connections between occupational, social and symptomatic function; neurocognitive ability and functional mental capacities. We argue that it logically follows that evidence of restoration of functional mental capacity itself can be viewed as a measure of recovery. When one considers the results shown by Naughton et al. [26], these findings highlight the need for future research into the relationship between mental capacity and recovery, along with informing potential directions for future therapeutic interventions. We now propose that this measure should be tested for sensitivity to change and as a treatment outcome measure. We plan to examine these hypotheses.

## Abbreviations

An: Attention
CI: Confidence Interval
DCL: Dundrum Capacity Ladders
FA: Finances Appreciation score
FD: Finances Decision score
FR: Finances Reasoning score
FT: Finances Total score
FU: Finances Understanding score
GAF Occ: GAF Occupational score
GAF Soc: GAF Social score
GAF Sym: GAF Symptom score
HA: Healthcare Appreciation score
HD: Healthcare Decision score
HR: Healthcare Reasoning score
HT: Healthcare Total score
HU: Healthcare Understanding score
MC: Matrics Composite
MIRECC-GAF: Mental Illness Research, Education and Clinical Center Global Assessment of Functioning
NCC: Neurocognitive Composite
PS: Processing Speed
Rg: Reasoning
SC: Social Cognition
SPSS: Statistical Package for the Social Sciences
VeL: Verbal Learning
ViL: Visual Learning
WA: Welfare Appreciation score
WD: Welfare Decision score
WM: Working Memory
WR: Welfare Reasoning score
WT: Welfare Total score
WU: Welfare Understanding score
